# Identification of Potential Genes in Pathogenesis and Diagnostic Value Analysis of Partial Androgen Insensitivity Syndrome Using Bioinformatics Analysis

**DOI:** 10.3389/fendo.2021.731107

**Published:** 2021-11-18

**Authors:** Yajie Peng, Hui Zhu, Bing Han, Yue Xu, Xuemeng Liu, Huaidong Song, Jie Qiao

**Affiliations:** ^1^ Department of Endocrinology, Shanghai Ninth People’s Hospital, Shanghai Jiao Tong University School of Medicine, Shanghai, China; ^2^ Research Centre for Clinical Medicine, Shanghai Ninth People’s Hospital, Shanghai Jiao Tong University School of Medicine, Shanghai, China

**Keywords:** androgen insensitivity syndrome, RNA transcriptome, differentially expressed genes, reproduction, immune, metabolism

## Abstract

**Background:**

Androgen insensitivity syndrome (AIS) is a rare X-linked genetic disease and one of the causes of 46,XY disorder of sexual development. The unstraightforward diagnosis of AIS and the gender assignment dilemma still make a plague for this disorder due to the overlapping clinical phenotypes.

**Methods:**

Peripheral blood mononuclear cells (PBMCs) of partial AIS (PAIS) patients and healthy controls were separated, and RNA-seq was performed to investigate transcriptome variance. Then, tissue-specific gene expression, functional enrichment, and protein–protein interaction (PPI) network analyses were performed; and the key modules were identified. Finally, the RNA expression of differentially expressed genes (DEGs) of interest was validated by quantitative real-time PCR (qRT-PCR).

**Results:**

In our dataset, a total of 725 DEGs were captured, with functionally enriched reproduction and immune-related pathways and Gene Ontology (GO) functions. The most highly specific systems centered on hematologic/immune and reproductive/endocrine systems. We finally filtered out CCR1, PPBP, PF4, CLU, KMT2D, GP6, and SPARC by the key gene clusters of the PPI network and manual screening of tissue-specific gene expression. These genes provide novel insight into the pathogenesis of AIS in the immune system or metabolism and bring forward possible molecular markers for clinical screening. The qRT-PCR results showed a consistent trend in the expression levels of related genes between PAIS patients and healthy controls.

**Conclusion:**

The present study sheds light on the molecular mechanisms underlying the pathogenesis and progression of AIS, providing potential targets for diagnosis and future investigation.

## Introduction

Androgen insensitivity syndrome (AIS; OMIM#300068), one of the common causes of 46,XY disorder/difference of sex development (DSD), is estimated to affect one in 20,000–100,000 live births and accounts for 40%–80% of 46,XY DSD patients (1–4). This disorder is divided into three categories, ranging from complete feminization with external genitalia [complete AIS (CAIS)] to various degrees of undervirilization [partial AIS (PAIS)] and to male infertility and/or gynecomastia [mild AIS (MAIS)]. AIS is an X-linked recessive disease caused by inactivating mutations in the androgen receptor (AR) gene (Xq11-q12), resulting in complete or partial resistance to the physiological effects of androgen in 46,XY individuals (5).

AR is a nuclear receptor that belongs to the steroid hormone receptor family. By binding to AR, androgens, such as testosterone (T) and dihydrotestosterone (DHT), could translocate into the nucleus of androgen target cells and bind to androgen response elements (AREs), which would regulate the expression of target genes (6). The AR-mediated effects of androgens are well known to be normally key elements for achieving appropriate internal and external male sex differentiation in both embryonic and secondary sexual development. In addition, normal AR function enables many target tissues to respond to androgen signaling, including the development and maintenance of bone and muscle mass, production and maturation of immune cells and thymocytes, and regulation of metabolism (7). The expression of various target genes is also controlled by AR, which could regulate cell growth, biosynthetic processes, and metabolism. Moreover, the repair of DNA damage could be affected by androgen signaling by regulating gene expression and transcription-related processes (8). Therefore, defects in *AR* could compromise the virilization process, exhibiting variably impaired masculinization of external genitalia, spermatogenesis arrest, gynecomastia, sparseness or absence of pubic and axillary hair, increased risk of gonadal tumors, and abnormal sex hormone levels (9). However, this disorder also leads to abnormal symptoms of other systems in males: slightly increased height as compared with normal females, disorder of skeletal development, inherited X-linked neurodegenerative disease, increased incidence of insulin resistance and cardiovascular risk, and risk of infection and autoimmune diseases were reported in CAIS and aromatase knockout (ARKO) mice (7, 10–13). However, except for *AR* mutation, the mechanisms underlying the genetic etiology of AIS have rarely been investigated.

The clinical diagnosis of AIS, especially PAIS, still represents a demanding challenge due to the phenotypes overlapping with 46,XY DSDs caused by other etiologies. Current evaluations rely mainly on clinical and biochemical features, karyotype analysis, *AR* genotype analysis, and functional studies of external genital skin-derived fibroblasts to detect the expression and binding capacity of AR receptors and androgen-regulated apolipoprotein D (APOD) (1). Pathogenic mutations of *AR* themselves result in lasting changes in gene expression in genital fibroblasts (14). However, the invasive procedure restricts the widespread clinical application of direct evidence derived from external genital skin fibroblasts. Moreover, in some patients with typical AIS phenotypes and impaired function of AR in the study of fibroblasts, no *AR* mutation was identified (14).

In this study, we performed comprehensive transcriptomic profiling of peripheral blood mononuclear cells (PBMCs) from genotype-proven PAIS patients and normal controls *via* RNA-seq analyses and focused on the differentially expressed genes (DEGs) of pubertal sex differentiation and fertility in males. The mechanism of AR defects was investigated from the potential genes that had never been studied in connection to AIS previously. Our results, linking the clinical features and molecular basis, contribute to the understanding of the mechanisms underlying the pathogenesis of AIS and provide new insights for convenient and economical measures that are helpful for clinical screening of AIS.

## Materials and Methods

### Subjects

The peripheral blood of three PAIS patients and three healthy male volunteers (age-matched) was obtained from Shanghai Ninth People’s Hospital (Shanghai Jiao Tong University School of Medicine, Shanghai, China). All the patients presented with micropenis, hypospadias, cryptorchid, and elevated serum testosterone levels. The external masculinization score (EMS) index was applied to describe the degree of undervirilization of the patients. Genetic analysis was performed by targeted next-generation sequencing (NGS), and two missense mutations (p.V686A and p.R761W) and a very rare synonymous mutation p.S889S [an exonic splicing mutation (15)] of the *AR* gene were identified in three AIS patients (**Table 1**). The variations were judged as pathogenic according to American College of Medical Genetics and Genomics (ACMG) guidelines, consistent with the classical phenotype, including micropenis, hypospadias, bifid scrotum, and cryptorchid. All subjects provided written informed consent. The Ethics Committee of the Shanghai Ninth People’s Hospital reviewed and approved this study.

**Table 1:** Clinical features, serum hormone, and *AR* mutations of three patients with PAIS.

	Patient 1	Patient 2	Patient 3
**Ages**	18	21	12
**Diagnosis**	PAIS	PAIS	PAIS
**Social gender**	F	M	M
**Clinical features**	MP, H, BS, BC	MP, H	MP, H, BS, BC
**Gynecomastia**	Yes	Yes	Yes
**EMS**	2	7	2
**Family history**	No	No	No
**FSH (mIU/ml)**	7.79	4.82	2.46
**LH (mIU/ml)**	29.33	11.67	5.91
**T (ng/ml)**	11.69	14.26	2.72
**AR mutation cDNA**	c.2667 C>T	c.2057 T>C	c.2281 A>T
**AR mutation protein**	p.S889S	p.V686A	p.R761W

Ages, age at first visit; PAIS, partial androgen insensitivity syndrome; M, male; F, female; MP, micropenis; H, hypospadias; BS, bifid scrotum; UC, unilateral cryptorchid; BC, bilateral cryptorchid; EMS, external masculinization score; FSH, follicle-stimulating hormone; LH, luteinizing hormone; T, testosterone.

### Isolation of Peripheral Blood Mononuclear Cells and RNA-seq Library Preparation

Five hundred microliters of saturated ethylenediaminetetraacetic acid (EDTA) was diluted with 50 ml of phosphate-buffered saline (PBS). The separated anticoagulated plasma samples were transferred into 50-ml centrifuge tubes and mixed with EDTA–PBS up to 50 ml. Along the walls of new 50-ml centrifuge tubes, the plasma–EDTA–PBS mixtures were carefully added to 15 ml of Lymphoprep (STEMCELL Technologies, Vancouver, Canada), a density gradient medium. PBMCs were centrifuged through the Lymphoprep layer during centrifugation at 1,200 *g* for 20 min at 4°C and then washed with EDTA–PBS. Total RNA was extracted from PBMCs by using TRIzol reagent (Invitrogen, USA) for RNA-seq. Single-end reads of 50 bp were used to sequence the libraries on the Illumina HiSeq platform (Igenecode Technology). For data analysis, Cufflinks (version 2.2.1) was used to annotate the fragment per kilobase of exons per million mapped reads (FPKM) against GENCODE (Release M22, GRCm38. p6).

### Differential Expression Analysis

To identify the DEGs between PAIS patients and normal males, gene expression profiles were compared by using the R-Bioconductor package limma. Student’s t-tests were used to calculate *p*-values. The DEGis were screened out by the following selection criteria: |log_2_ fold change (FC) > 1|, and the threshold value of the *p*-value was 0.05.

### Functional Enrichment Analysis of Differentially Expressed Genes

The functional categories Kyoto Encyclopedia of Genes and Genomes (KEGG) and Gene Ontology (GO) enrichment analyses of DEGs were performed by using Metascape (http://metascape.org/gp/index.html) (16). Functional enrichment research on large scales is often conducted through GO analysis, in which gene functions are categorized as biological process (BP), molecular function (MF), and cellular component (CC). DEGs were assigned to specific pathways using KEGG pathway analysis, which enabled the construction of reaction, molecular interaction, and relationship networks (17). The following criteria in Metascape were used to obtain a significant difference: minimum count of 3, enrichment factor of >1.5, and pick selective GO clusters. *p* < 0.05 was considered statistically significant.

### Protein–Protein Interaction Network Analysis and Gene Cluster Identification

The Search Tool for the Retrieval of Interacting Genes (STRING; http://string-db.org) (version 10.0) online database was used to predict the protein–protein interaction (PPI) network between proteins encoded by the top 236 DEGs (|log_2_ FC > 1.5|) and other proteins. PPI pairs with a combined score >0.4 were extracted. Subsequently, a PPI network was generated using Cytoscape Software (http://cytoscape.org/). We used the Molecular Complex Detection (MCODE) plugin to perform gene network clustering analysis, which identified the most significant modules within PPI networks with the standard degree cutoff = 2, node score cutoff = 0.2, k-core = 2, and maximum depth = 100.

### Hub Gene Selection and Analysis

The degree of each protein node was calculated, and the top 10 genes identified as hub genes were picked according to the CytoHubba plugin of Cytoscape. The Cytoscape plugin Biological Networks Gene Oncology tool (BiNGO) (version 3.0.3) was used to visualize the BP analysis of the top five hub genes (degree >10) (18). The color depth of nodes represents the corrected *p*-value of ontologies, and the size of nodes indicates the numbers of genes that are contained in the ontologies. *p* < 0.01 indicated a significant difference.

### Tissue-Specific Gene Expression Analysis

The tissue-specific expression of the DEGs was analyzed by using the online resource BioGPS (http://biogps.org). The inclusion criteria were as follows: 1) expression level of >10 times the median for transcripts that mapped to a single tissue, and 2) the second highest expression level was no more than 1/3 as high as the highest level (19).

### Transcription Factor Analysis

Epigenetic landscape *in silico* detection (LISA) was used for transcription factor (TF) analysis (20). The upregulated and downregulated DEGs were entered into the gene lists to analyze the key TFs contributing to DEGs. TFs ranking lists and volcano plots were created automatically.

### Quantitative Real-Time PCR

Reverse transcription was conducted using the PrimeScript RT Master Mix Kit (Takara, Dalian, China). The mRNA level was assessed using TB Green Premix Ex Taq Kit (Takara, Dalian, China) following the instructions. Primers are available in **Supplementary Table 4**. The relative expression of mRNA was calculated by the 2^−ΔΔCt^ method with normalization to GAPDH.

## Results

### Differential Expression Analysis

**Figure 1:**
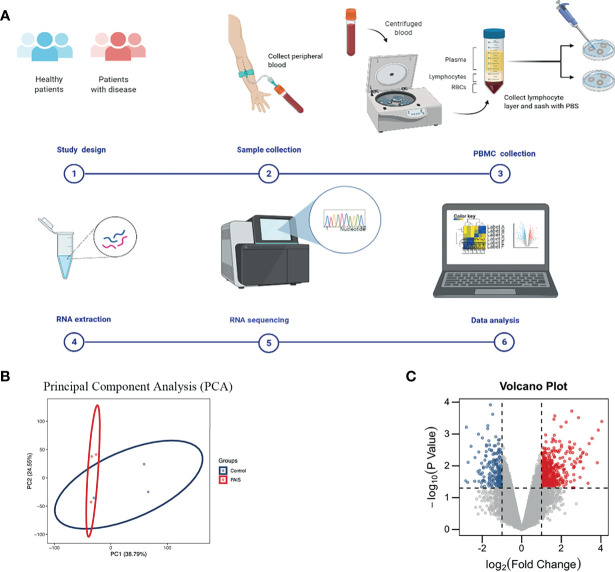
Experiment process and principal component analysis (PCA) and volcano plot of differentially expressed genes (DEGs). **(A)** Schematic diagram of experiment procedure. **(B)** PCA of two groups. **(C)** DEGs in partial androgen insensitivity syndrome (PAIS) patients and healthy controls samples are shown in the volcano plot, with blue dots representing significantly downregulated genes and red dots representing significantly upregulated genes.

**Figure 2:**
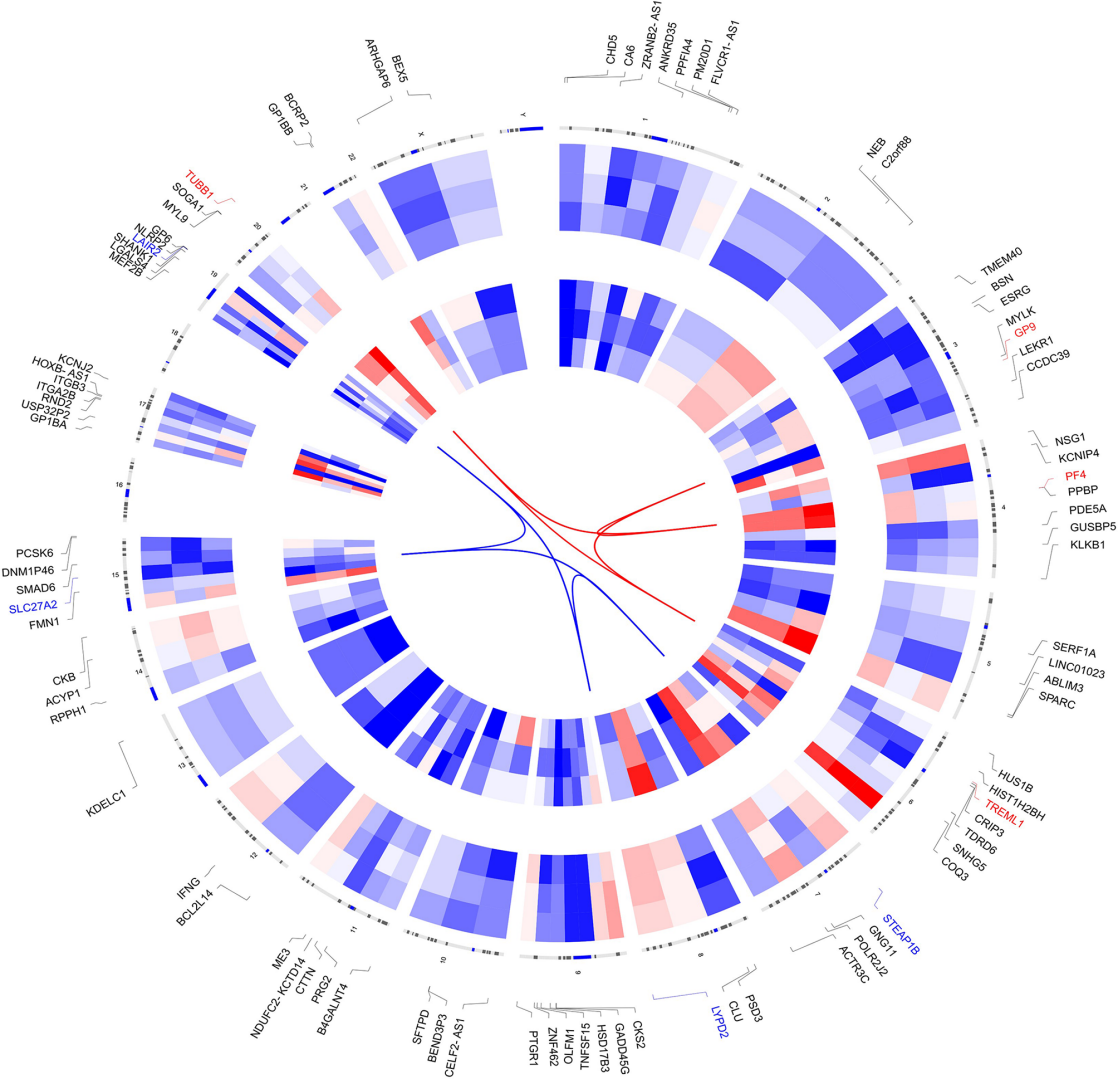
Circos plot of expression patterns and chromosomal positions of top 100 differentially expressed genes (DEGs). The outer circle represents chromosomes, and lines coming from each gene point to their specific chromosomal locations. The group of patients is represented in the inner circular heatmaps. According to |log_2_ fold change|, the top four upregulated genes (red) and the top four downregulated genes (blue) are connected with red and blue lines in the center of the Circos plot.

The clinical features, serum hormones, and *AR* mutations of the three cases are summarized in **Table 1**. To determine the transcriptomic variance among the PAIS patients and normal controls, RNA-seq was carried out on each sample to obtain the transcriptome profiles (**Figure 1A**). Following the elimination of adapter sequences and low-quality reads, signals of 14,318 transcripts were obtained from the six samples. Groups were well discriminated from patients and normal in a principal component analysis (PCA) model, as shown in **Figure 1B**. Based on the criteria of |log_2_ FC > 1| and *p* < 0.05, we observed that PBMCs of patients exhibited significantly different gene expression patterns (**Figure 1C**). In **Figure 2**, according to |log_2_ FC|, we chose the top 100 DEGs to visualize their expression patterns, chromosomal locations, and logarithmic adjusted *p*-value shown in the inner layer. Compared with those in normal individuals, a total of 725 DEGs, including 495 upregulated and 230 downregulated genes, were identified in PAIS patients (**Supplementary Table 1**).

### Functional Enrichment Analysis of Differentially Expressed Genes

**Figure 3:**
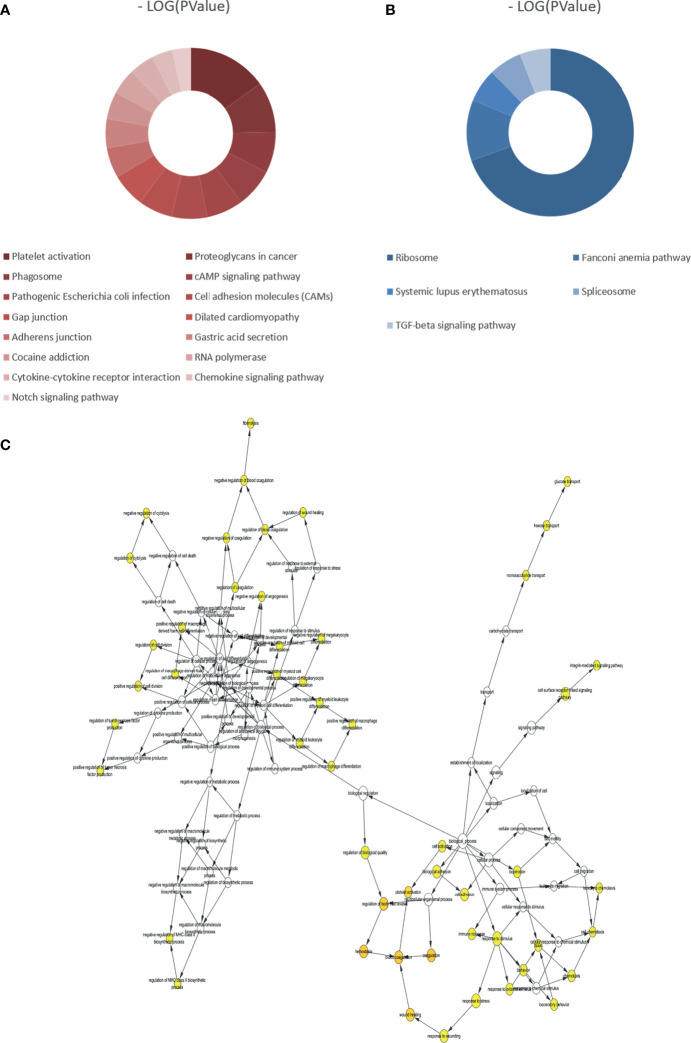
Kyoto Encyclopedia of Genes and Genomes (KEGG) analyses of all differentially expressed genes (DEGs) and biological process analysis of the hub genes (degree >10). **(A)** KEGG pathways of upregulated DEGs. **(B)** KEGG pathways of downregulated DEGs. **(C)** The biological process analysis of hub genes (degree >10). The color depth of nodes refers to the corrected *p*-value of ontologies. The size of nodes refers to the numbers of genes that are involved in the ontologies.

**Figure 4:**
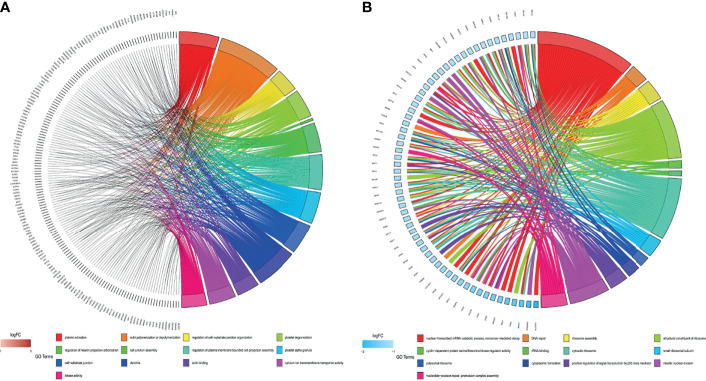
Gene Ontology (GO) analyses of all differentially expressed genes (DEGs). **(A)** GO enrichment of the upregulated DEGs. **(B)** GO enrichment of the downregulated DEGs.

According to the *p*-value <0.05, 15 and five KEGG pathways of the DEGs with upregulation and downregulation respectively were screened out, and the pie chart was drawn according to the enrichment score (**Figures 3A, B**). The enriched signaling pathways with upregulated DEGs included platelet activation, proteoglycans in cancer, and chemokine signaling pathways. The highly expressed genes were related to sex differentiation and cell development signaling pathways, such as phagosome, cAMP, notch, and adherens junction. The downregulated DEGs were significantly enriched in ribosome, TGF-β. For GO, the top 13 of these terms based on their *p*-value are shown in chord plots (**Figure 4**). The DEGs were enriched mainly in BPs, with upregulated DEGs specifically centered on platelet activation, actin polymerization or depolymerization, regulation of cell-substrate junction organization, platelet degranulation, regulation of neuron projection arborization, cell junction assembly, etc. The majority of the downregulated DEGs were related to DNA repair, gene expression, meiotic nuclear division, and apoptosis. Regarding MF, actin binding, calcium ion transmembrane transporter activity, and kinase activity were the most remarkably enriched GO terms in the upregulated DEGs, while structural constituent of ribosome, cyclin-dependent protein serine/threonine kinase regulator activity, and rRNA binding were mainly enriched in the downregulated DEGs.

### Protein–Protein Interaction Network Analysis and Gene Cluster Identification

**Figure 5:**
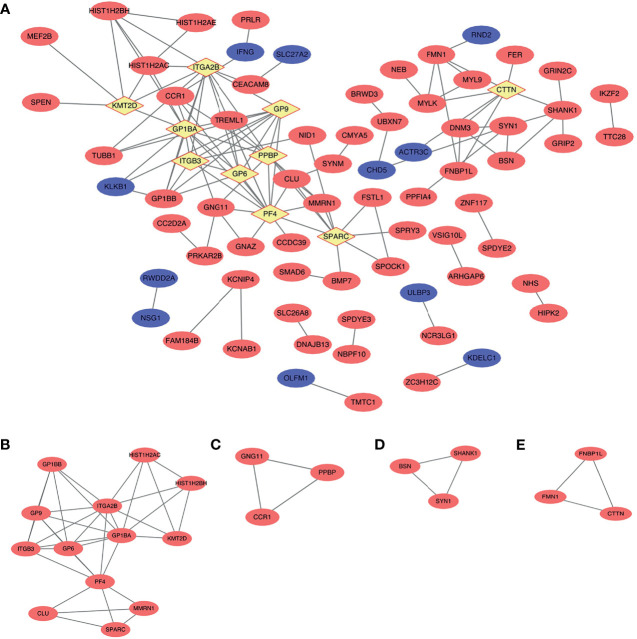
Protein–protein interaction (PPI) network and the key modules of top 236 differentially expressed genes (DEGs) (|log_2_ (fold change) >1.5|). **(A)** Cytoscape network visualization of the 182 nodes and 123 edges that were obtained with interaction scores >0.4 according to the STRING online database. The nodes denote proteins, and the edges correspond to the interactions between two proteins. Red, blue, and yellow colors represent upregulated, downregulated, and hub genes, respectively **(B–E)** Four key modules were identified by MCODE.

According to the criteria of |log_2_ FC > 1.5|, 182 nodes and 123 edges comprised the PPI network; the network had an interaction score >0.4 and PPI enrichment *p*-value < 1.91e−07 based on the STRING online database (**Figure 5A**). We identified four key modules with upregulated genes by MCODE to further analyze the PPI network of DEGs (**Figures 5B–E**, **Table 2**). Genes in the first gene module with the highest score were primarily related to gene silencing and platelet- and immune system-related functions. Furthermore, the results showed that genes in the other three modules were enriched mainly in methylation, cell adhesion synaptic transmission, and inflammatory response.

**Table 2:** MCODE was used to process the data downloaded from the STRING to further mine gene clusters.

Cluster	Score (Density*#Nodes)	Nodes	Edges	Node IDs
1	5.833	13	35	CLU, GP1BB, ITGB3, ITGA2B, GP9, SPARC, KMT2D, PF4, HIST1H2AC, HIST1H2BH, GP1BA, MMRN1, GP6
2	3	3	3	GNG11, PPBP, CCR1
3	3	3	3	BSN, SYN1, SHANK1
4	3	3	3	FNBP1L, FMN1, CTTN

Specific data of gene clusters were exported and presented in a tabular form.

### Hub Gene Selection and Analysis

In the PPI network, the top 10 genes that were evaluated by connectivity degree were considered hub genes for subsequent analysis, including PF4, GP1BA, ITGA2B, GP9, PPBP, SPARC, GP6, ITGB3, CTTN, and KMT2D. The BP analysis of the top five hub genes (degree >10) is shown in **Figure 3C**, and these genes were found to be principally involved in coagulation, cell adhesion, cytolysis, cell division, and immune response. Many hub genes, such as GP1BA, GP9, ITGA2B, ITGB3, PPBP, and PF4, were found to be key genes enriched in the chemokine signaling pathway, focal adhesion, extracellular matrix (ECM)–receptor interaction, and PI3K–Akt signaling pathway within each breed.

### Tissue-Specific Gene Expression Analysis

BioGPS identified 75 genes among the top 234 DEGs (cutoff of |log_2_ FC > 1.5|) that were expressed in a particular tissue or organ system (**Supplementary Table 2**). The most highly represented systems were hematologic/immune and reproductive/endocrine systems, which accounted for 57.3% of the total tissue-specific expression genes. The neurologic and muscle systems had a relatively low percentage, which was 18.6% (14/75) and 8% (6/75) of tissue-specific genes, respectively, while the urinary and digestive systems had similar levels of enrichment (2.6%, 2/75). In addition, the circulatory and fat systems had the lowest enrichment levels (1.3%, 1/75) (**Table 3**).

**Table 3:** Tissue-specific expressed genes identified by BioGPS.

System	Genes
Hematologic/immune	CKS2, ANKRD35, LAIR2, REL, SNHG5, SMIM24, CCR1, MMRN1, HIST1H2AC, IFI44L, PRKAR2B, ITGA2B, NBPF10, CEACAM8, PRG2, MEF2B, KCNJ2, GP1BB, PPBP, ITGB3, PF4, GP9
Neurologic	OLFM1, CHD5, NSG1, FNBP1L, MFAP3L, BSN, USP32P2, PSD3, DNM3, SYN1, GNAZ, KCNAB1, CLU, HIPK2
Respiratory	PTGR1, SMAD6, DST, SFTPD
Muscle	FSTL1, SPARC, CMYA5, SPOCK1, NEB, GNG11
Digestive	PCSK6, LGALS4
Circulatory	CTTN
Reproduction/Endocrine	CENPS, IQCK, TSSK2, TTC28, SYNM, SLC26A8, MYL9, HSD17B3, MYLK, CKB, ARHGAP6, HIST1H2BH, ZFHX3, PLCD4, SLC5A3, BMP7, BEX5, PPFIA4, AANAT, GADD45G, CA6
Placenta	COLEC12, ESAM
Fat	NID1
Urinary	KCNJ1, SLC27A2

### General Analysis of Transcription Factors

**Figure 6:**
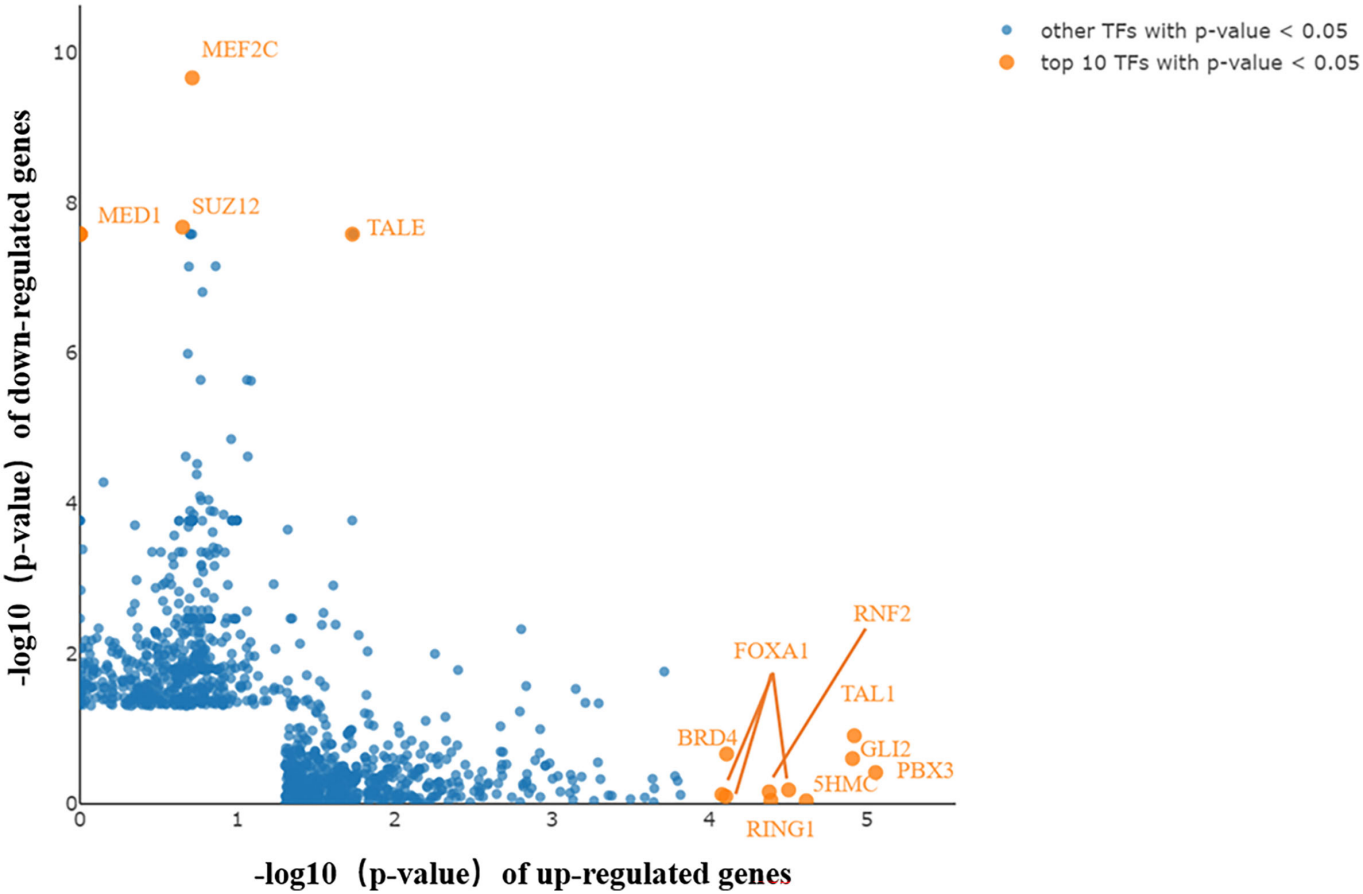
Key transcription factors (TFs) generated by Lisa analysis. The top 10 TFs are displayed in orange and marked with gene symbols beside them. Key TFs potentially contributing to the differentially expressed genes (DEGs) in upregulated and downregulated genes.

TFs bind to specific DNA sequences to regulate gene expression activation or repression. Coactivators and TFs interact to control the spatial and temporal expression of genes (21). LISA TF analysis was performed to identify the TFs that caused differential gene expression in PAIS. Following inputting selected genes for analysis, key TFs were subsequently generated (**Figure 6**). The top 10 upregulated and four downregulated candidates may participate in the genesis and progression of PAIS, including RNF2, FOXA1, TAL1, BRD4, GLI2, 5HMC, PBX3, RING1 and TALE, SUZ12, MED1, and MEF2C.

### Potential Genes Related to Disorder of Sex Development

**Figure 7:**
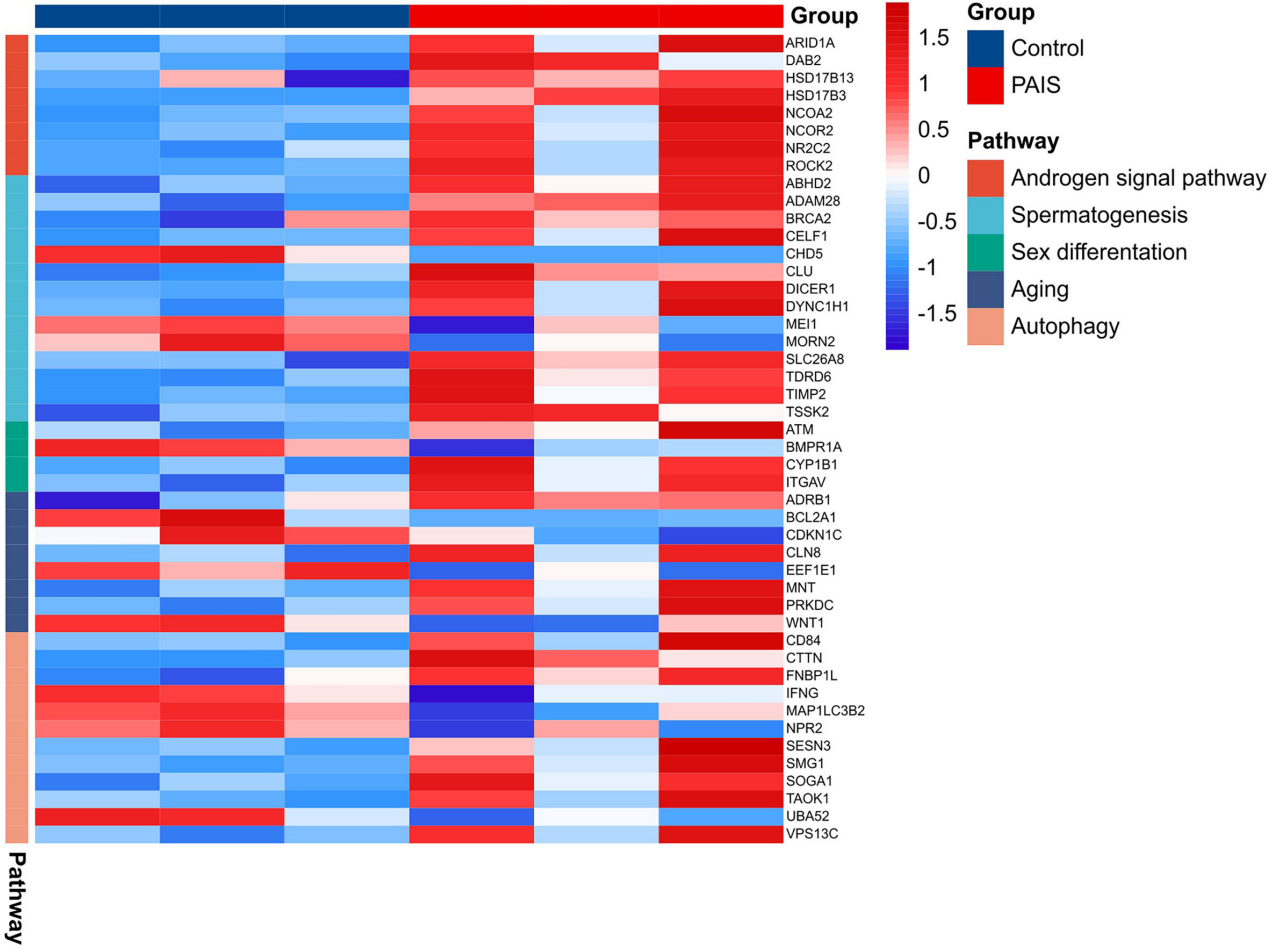
A heatmap of possible related genes in sex development.

The results revealed that the gene expression profiles were significantly disturbed in patients with PAIS compared with normal controls. This disturbance is especially visible in genes closely related to sex development (**Figure 7**). In our analysis, we distinguished three downregulated DEGs (MEI1, CHD5, and MORN2) in connection to spermatogenesis, spermatid development, and spermatid differentiation (**Supplementary Table 3**). ACTR3C, ITGA7, and ATF4 were involved in Sertoli cell function, whereas HSD17B3 and CYP1B1 participated in testosterone metabolic processes. Androgen signaling pathway including important transcriptional coactivators and corepressors, such as NCOA2, ARID1A, DAB2, NCOR2, ROCK2, and NR2C2, also showed significant differences. Several genes were associated mostly with sex differentiation, aging, and cell development. In addition, we also observed significant transcriptome differences in autophagy between patients and controls.

### Identification of Genes of Interest

MCODE analysis detected key clusters consisting of 22 genes (**Table 2**). Combined with the tissue-specific gene expression analysis, we identified genes of interest that were particularly upregulated in the hematologic/immune system (CCR1, PPBP, and PF4) and the metabolism/endocrine system (GP9 and SPARC). Additionally, we manually identified two additional genes of interest (CLU and KMT2D) that are potentially involved in the pathogenesis of PAIS by the GeneCards database. The abbreviations, names, and functions for these 22 genes of interest are shown in **Table 4**.

**Table 4:** Functional roles of 22 genes of interest.

Gene symbol	Full name	Fold change	Function
**Hematologic/immune system**			
CCR1	C-C motif chemokine receptor 1	3.008	CCR1 causes inflammation and cell infiltration
PPBP	Pro-platelet basic protein	8.422	PPBP is associated with development or progression of multiple autoimmune diseases
PF4	Platelet factor 4	10.611	PF4 protein functions as an inhibitor of T-cell proliferation, angiogenesis, and hematopoiesis
ITGB3	Integrin subunit beta 3	8.622	ITGB3 is known to participate in cell adhesion as well as cell surface-mediated signaling
GP9	Glycoprotein IX platelet	16.516	GP9 mediates platelet adhesion to blood vessels
GP6	Glycoprotein VI platelet	5.348	GP6 is essential for the formation of arterial thrombosis
GP1BB	Glycoprotein Ib platelet beta subunit	7.940	GP1BB participates in the formation of platelet plugs by binding to von Willebrand factor
ITGA2B	Integrin subunit alpha 2b	5.746	This receptor plays a crucial role in the blood coagulation system, by mediating platelet aggregation
MMRN1	Multimerin 1	2.997	It may function as an extracellular matrix or adhesive protein
GP1BA	Glycoprotein Ib platelet alpha subunit	5.762	GP1BA leads to enhanced platelet activation, thrombosis, and hemostasis
GNG11	G protein subunit gamma 11	7.914	GNG11 suppresses cell growth and regulates cellular senescence
**Metabolism system**			
SPARC	Secreted protein acidic and cysteine rich	6.296	SPARC is implicated in the pathogenesis of metabolic syndrome
KMT2D	Lysine methyltransferase 2D	3.354	KMT2D is related to obesity, lipid accumulation, glucose tolerance, and insulin sensitivity
HIST1H2AC	Histone cluster 1 H2A family member c	3.032	HIST1H2AC is associated with cardiovascular risk factors, e.g., T2DM, and diseases, including myocardial, infarction, heart failure, and atherosclerosis
**Reproduction system**			
CLU	Clusterin	5.948	CLU thought to be concerned with improving spermatogenesis
HIST1H2BH	Histone cluster 1 H2B family member h	6.173	Among its related pathways are DNA double-strand break response and cellular senescence
**Circulatory system**			
CTTN	Cortactin	7.735	CTTN contributes to the organization of the actin cytoskeleton and cell shape
**Neurologic system**			
FNBP1L	Formin binding protein 1 like	2.885	It may be involved in autophagy
SYN1	Synapsin I	3.400	This member of the synapsin family plays a role in regulation of axonogenesis and synaptogenesis
BSN	Bassoon presynaptic cytomatrix protein	7.307	BSN is essential for regulating neurotransmitter release from a subset of synapses
FMN1	Formin 1	4.960	FMN1 lays a role in the formation of adherens junction and the polymerization of linear actin cables
SHANK1	SH3 and multiple ankyrin repeat domains 1	6.121	It acts as scaffold proteins that are required for the development and function of neuronal synapses

### Validation of the Differentially Expressed Genes of Interest in Partial Androgen Insensitivity Syndrome Patients

**Figure 8:**
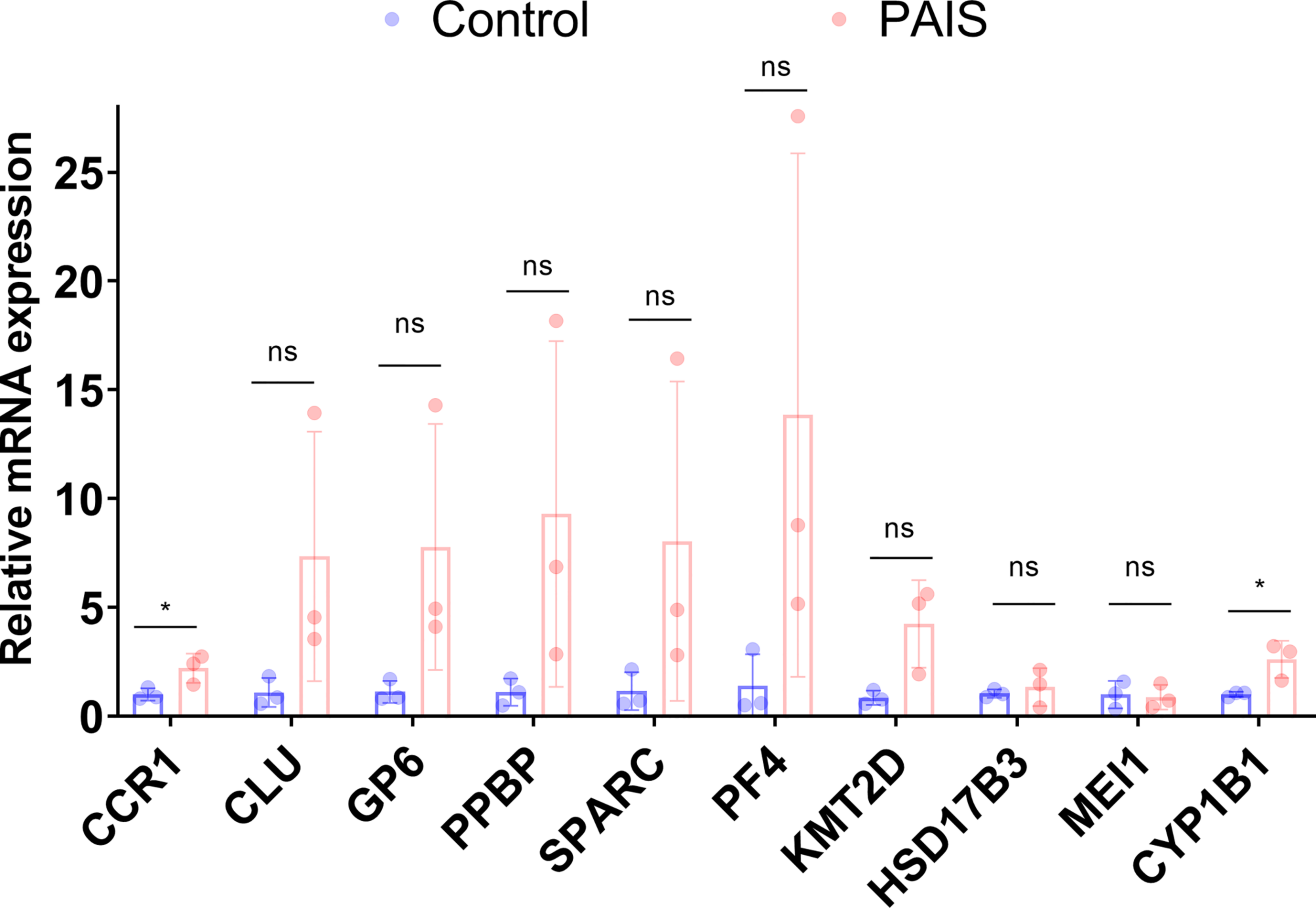
RNA expression of genes of interest were measured in partial androgen insensitivity syndrome (PAIS) and healthy samples. RNA expression of CCR1, PPBP, PF4, CLU, KMT2D, GP6, SPARC, CYP1B1, HSD17B3, and MEI1 was measured. *p*-Values were calculated using a two-sided unpaired Student’s t-test. *p < 0.05; ns, not significant.

The selected biomarkers were validated in PBMCs of PAIS using qRT-PCR analysis. Consistent with the results of the mRNA microarray, the expression level of CCR1 was obviously higher in PAIS patients than in controls. However, the expression levels of PPBP, PF4, CLU, KMT2D, GP6, and SPARC showed the same trends as the bioinformatics analysis results, although there was no significant statistical difference between the two groups due to the limited number of samples (**Figure 8**). Notably, the expression levels of the above target genes were the lowest in the PBMCs of patient 1 with synonymous mutation p.S889S, suggesting relatively mild impairment of this variation with a small fraction of normal AR transcripts. In addition, the expression levels of CYP1B1, HSD17B3, and MEI1, which are involved in the androgen signaling pathway, were also changed (**Figure 8**).

## Discussion

AIS is a rare endocrine disorder falling within the category of 46,XY DSD caused by mutations within the *AR* gene located on the X chromosome long arm and is classified as CAIS, PAIS, or MAIS (22). PAIS, a relatively frequent type, has a variable clinical presentation depending on the degree to which the external genitalia responds to androgens (23, 24). Psychological distress occurred due to the vague gender identity of the patients (25). The risk of germ cell tumors seems to be higher in patients with PAIS than in patients with CAIS (26). Therefore, it is urgent to reveal potential markers that are helpful for accurate diagnosis and prompt treatment. However, effective biomarkers for diagnosis remain scarce, as well as the reasonable predictors for androgen therapy, which actually are important for sex assignment accordingly (27). To our knowledge, for the first time, we investigated the variance of gene expression profiles of PBMCs in genotyping proven PAIS by RNA-seq and identified potential hub genes as candidate predicted factors for the disorder.

AR and the androgen–AR signaling pathway play significant roles in sexually dimorphic development and the function of male reproductive and non-reproductive organs, such as sex development, spermatogenesis, regulation of growth and metabolism, muscle mass/function, bone metabolism, and certain aspects of the immune and cardiovascular system (28). More attention has been drawn to the impaired function of AR in sexual development, but defects in other regulatory processes or systems have long been neglected. Our results are consistent with previous limited studies, providing reasonable support from different aspects. In this study, we identified 725 DEGs, including 495 upregulated and 230 downregulated genes, in the PBMCs of PAIS patients compared with age-matched 46,XY controls. The upregulated genes screened out by combined analysis were mostly enriched in platelet activation, proteoglycans in cancer, chemokine signaling pathway, phagosome, cAMP, notch, and adherent junction, while the downregulated genes were enriched mainly in ribosome and TGF-β signaling pathway. Notably, proteoglycans and their modifying enzymes have been implicated in tumorigenesis in numerous cancers, including germ cell tumors (29–31). Moreover, previous studies have reported that adhesion-related genes, which are involved in cytoskeleton remodeling, actin dynamics, integrin signaling, and focal adhesion formation, were upregulated after suppression of the action of both testosterone and follicle-stimulating hormone (FSH) by facilitating aggregation/clumping of round spermatids together and formation of giant multinucleated germ cells (32, 33). Cell adhesion was also related to sexual immaturity (34). Additionally, phagosome, cAMP, TGF-β, and notch signaling pathways play crucial roles in sexual differentiation and the maintenance of gonad function (35–38). Furthermore, genes from the key modules were related mostly to immune function, including the chemokine signaling pathway, focal adhesion, ECM–receptor interaction, and PI3K–Akt signaling pathway. GO enrichment analysis showed that reduced expression was primarily enriched in DNA repair, translation, and meiotic division processes, which are closely correlated with development and spermatogenesis.

According to our data, the top TFs of upregulated genes (e.g., RNF2, FOXA1, BRD4, Gli2, 5HMC, and PBX3) and downregulated genes (e.g., TALE and MEF2C) were identified, most of which have been indicated to participate in regulating sex development (39–42). FOXA1 has been reported to act as a pioneer factor that opens up compact chromatin to facilitate the recruitment of AR. The expression of FOXA1 was observed to cooperate and imbalance the AR cistrome in regulating AR signaling in prostate cancer cells, which may form a mechanism compensating for the functional defects of AR, even in PBMCs with a low abundance of AR expression (43). Recent studies have demonstrated that Gli2 is a main regulatory gene mediating the hedgehog signaling pathway, which not only is a crucial factor for initial development but also plays a vital role in coordination with androgen signaling and in sexually dimorphic development of the external genitalia (44). Therefore, the alteration of Gli2 may represent a regulatory or compensatory mechanism in this disease, underlying the mechanism of hypospadias. Unlike other TFs, MEF2C plays a crucial role in myogenesis of cardiac, skeletal, and smooth muscle and is essential for the differentiation of muscle (45, 46). Current literature on PBX3 is known to be a functionally significant TF in a range of cancers, including prostate cancer, and linked to shorter overall survival; and the miR inhibitor of PBX3, miR-let-7d, is regulated by androgen (47). Using our preliminary analysis, we prioritized TFs that were likely to play a role in the pathophysiology of PAIS.

Then, BioGPS showed that hematologic/immune and reproductive/endocrine factors were significantly dysregulated, elucidating the common occurrence of immune dysfunction and sexual abnormalities in PAIS patients. The overexpression of immune system genes in the patient transcriptome may be a possible early indication of autoimmune disorders that could be involved in the onset of autoimmune diseases seen in AIS patients, including celiac disease (48) and primary and secondary Sjögren’s syndrome (49). Similarly, a higher prevalence of autoimmune diseases has been suggested in patients with male hypogonadism (50). Previous studies have shown that androgens have a suppressive effect on the immune system and play a key role in the development of immune cells (7). The TGF-β pathway was proven to regulate immune tolerance and homeostasis against self- and microbe antigens (51), also appearing in our functional enrichment results.

Combined BioGPS and PPI key module analyses discovered a number of novel genes that have been previously not been known to be related to this disorder. Three selected genes of interest (CCR1, PPBP, and PF4) were associated with the hematologic/immune system, among which PPBP and PF4 served as the hub genes. In an original study, CCR1 was shown to be a G protein-coupled receptor expressed by various leukocyte types that leads to inflammation and activation of robust cell infiltration, so it is considered to be an appropriate target for autoimmune and inflammatory therapeutics (52). Intriguingly, PPBP (CXCL7) and PF4 (CXCL4) belong to the CXC chemokine family, which plays a pivotal role in the development or progression of rheumatoid arthritis (RA), antiphospholipid syndrome, and systemic lupus erythematosus (53, 54). The level of PPBP was found to be significantly higher in vasculitis patients and female RA patients than in ordinary people (55, 56). PF4 protein functions as a chemoattractant for multiple types of cells, as well as an inhibitor of T-cell proliferation, angiogenesis, and hematopoiesis. Thus, we speculated that certain interrelations exist between AIS and the immune system. However, clinical data and basic research on these issues are scarce, and the immune response varies in knockout mice (T-ARKO or Tfm), which may not be comparable with AIS patients because of orchidectomy and supplementation with E2. In summary, whether AIS patients are susceptible to autoimmune diseases, inflammation, and infectious diseases should be studied further.

Impaired AR signaling can be responsible for an increased risk of metabolic diseases (10, 57, 58). The genes in reproduction/endocrine metabolism we found were CLU, KMT2D, GP6, and SPARC. CLU, thought to be involved in spermatogenesis, encoding clusterin, may increase in non-capacitated sperm to play a protective role in mice and bovines (59, 60). The protein encoded by KMT2D is histone H3 lysine 4-methyltransferase, which regulates muscle and adipose tissue development. Heterozygous Kmt2d+/− mice demonstrated improved obesity, lipid accumulation, glucose tolerance, and insulin sensitivity (61). GP6, as a new acute coronary syndrome marker encoding platelet glycoprotein VI, is essential for the formation of arterial thrombosis (62). SPARC, a profibrotic protein secreted by adipocytes, is implicated in the pathogenesis of inflammation, dyslipidemia, increased cardiovascular risk, obesity, and type 2 diabetes (63). These data indicated that dysregulation of KMT2D, GP6, and SPARC might be responsible for the increased risk of metabolic syndrome, diabetes, and cardiovascular diseases in AIS.

By manual screening of these DEGs, we showed that some robust genes were disordered in male reproductive development, such as spermatogenesis, sex differentiation, autophagy, aging, and androgen signaling pathways. Among these genes, CYP1B1, an enzyme belonging to the cytochrome P450 superfamily, has roles in the masculinization process that are regulated by androgens. During genital tubercle masculinization, CYP1B1 may function to metabolize androgens, such as testosterone. Consistent with Tanase-Nakao et al. (64), who reported in the transcriptomics of external genital fibroblasts of PAIS patients, CYP1B1 was also found to be elevated in the PBMCs of patients. As a potential androgen target gene, CYP1B1 may gain intriguing insights from the current study for new biomarkers in the diagnosis of AIS.

HSD17B3 encodes hydroxysteroid 17-beta dehydrogenase 3, which catalyzes the conversion of the more biologically active testosterone in the testis and is required for normal fetal development of the male genitalia. HSD17B3 expression has been reported in the Sertoli cells of the gonads in CAIS patients, which preserves the characteristics of the fetal gonad in the postpubertal testis in androgen synthesis (65). For the upregulation under elevated androgen levels in PAIS patients, it could be assumed that the expression of HSD17B3 might be modulated by the AR-targeted gene but not the product testosterone. The downregulated genes CHD5 and MEI1, encoding chromodomain helicase DNA binding protein 5, which regulates the histone-to-protamine chromatin remodeling process, and meiosis inhibitor protein 1, which affects male meiosis, respectively, play a spermatogenetic role (66, 67).

Previous studies aimed to identify potential biomarkers for the process of germ cell differentiation by using testicular tissue from CAIS patients (68) and mined predictors of genital development and AR function in genital skin fibroblasts from PAIS patients (64). A few genes have been identified as candidates to decipher androgen-dependent normal and abnormal genital development by genital fibroblasts (14, 69). ApoD has not been identified as a DEG due to its low abundance, which may result from the specific expression profile in different tissues. The limitation of the study is the small sample size, only included in genotype-proven PAIS patients. Mutation-negative AIS should be investigated as a separate group in future work (70).

In our study, we first performed a system-wide study of PAIS using PBMCs, with different compositions of DEGs in the results due to the specific expression profile of different tissues. However, the common DEGs (CYP1B1 and HSD17B3) could also be identified in PBMCs and in genital skin fibroblasts of PAIS patients, which could be easy biomarkers for diagnosis. Our approach provides valuable information for reproductive-related molecular biomarkers for screening and casts light on the understanding of AR and the possible pathogenesis of AIS.

## Data Availability Statement

The original contributions presented in the study are publicly available. These data can be found here: https://www.ncbi.nlm.nih.gov/bioproject/PRJNA767615.

## Ethics Statement

The studies involving human participants were reviewed and approved by The Ethical Committee of the Shanghai Ninth People’s Hospital, China. The patients/participants provided their written informed consent to participate in this study.

## Author Contributions

YP performed the data analysis. YP and HZ contributed to the writing and revision of this manuscript. BH, YX, and LX enrolled the patients and sorted out the data. HS and JQ conceived and designed the experiments and revised the manuscript. All authors contributed to the article and approved the submitted version.

## Funding

This study was funded by the National Natural Science Foundation of China (grant no. 81873652), the Natural Science Foundation of Shanghai (grant no. 21ZR1438300), and Clinical Research Project of Multi-Disciplinary Team, Shanghai Ninth People’s Hospital, Shanghai Jiaotong University School of Medicine (201903).

## Conflict of Interest

The authors declare that the research was conducted in the absence of any commercial or financial relationships that could be construed as a potential conflict of interest.

## Publisher’s Note

All claims expressed in this article are solely those of the authors and do not necessarily represent those of their affiliated organizations, or those of the publisher, the editors and the reviewers. Any product that may be evaluated in this article, or claim that may be made by its manufacturer, is not guaranteed or endorsed by the publisher.
